# Breastfeeding and maternal eating behaviours are associated with child eating behaviours: findings from the ROLO Kids Study

**DOI:** 10.1038/s41430-020-00764-7

**Published:** 2020-09-30

**Authors:** Cara A. Yelverton, Aisling A. Geraghty, Eileen C. O’Brien, Sarah Louise Killeen, Mary K. Horan, Jean M. Donnelly, Elizabeth Larkin, John Mehegan, Fionnuala M. McAuliffe

**Affiliations:** 1 UCD Perinatal Research Centre, School of Medicine, University College Dublin, National Maternity Hospital, Dublin, Ireland; 2 UCD School of Public Health, Physiotherapy and Sports Science, University College Dublin, Belfield, Dublin 4, Ireland

**Keywords:** Translational research, Risk factors, Obesity, Nutrition

## Abstract

**Background:**

Child eating behaviours can negatively contribute to the development of childhood obesity. This study investigated associations between breastfeeding habits, maternal eating behaviours and child eating behaviours, in 5-year-old children.

**Methods:**

Secundigravida women were recruited to the ROLO dietary randomised controlled trial (Dublin, Ireland) and were followed up with their children to 5 years of age. Breastfeeding exposure and duration were obtained at postnatal and infant follow-up at 2 and 6 months and 2 and 5 years. At 5 years, maternal and child eating behaviours were measured using the Three Factor Eating Questionnaire and the Child Eating Behaviour Questionnaire, respectively. Regression determined associations between breastfeeding habits and maternal eating behaviours with child eating behaviours, controlling for RCT group, maternal education level, maternal BMI at 5 years, childcare exposure and child BMI centile at 5-year follow-up.

**Results:**

There were 230 mother and child pairs analysed. One hundred and fifty-eight mothers had initiated breastfeeding. Median breastfeeding duration was 22 (IQR 33) weeks. Children who were never breastfed were more likely to express a desire to drink (*B* = −1.01, *p* = 0.022). Longer breastfeeding duration was associated with lower food responsiveness (*B* = −1.71, *p* = 0.003). Maternal uncontrolled eating was positively associated with child food responsiveness, emotional overeating and emotional undereating (*B* = 0.21, *p* < 0.001; *B* = 0.14, *p* = 0.005; *B* = 0.14, *p* = 0.005, respectively). Maternal emotional eating was associated with child emotional overeating and undereating (*B* = 0.27, *p* < 0.001, *B* = 0.29, *p* = 0.004, respectively).

**Conclusion:**

Not breastfeeding and short breastfeeding duration may contribute to the development of obesogenic eating behaviours in children, alongside maternal eating behaviours including uncontrolled and emotional eating. These ‘food approach’ eating behaviours may increase risk of overweight/obesity as they are associated with increased energy intake, hence the importance of research surrounding eating behaviours.

## Introduction

Childhood obesity is a global issue. Ireland is no exception with one in five Irish children aged 5–12 years having overweight or obesity [1]. This indicates a minor improvement from the National Children’s Food Survey in 2003–2004 [2], at which point one in four children had overweight/obesity. However, the issue of obesity among Irish children remains and needs to be addressed.

Child eating behaviours are known to play a role in the aetiology of obesity. Child eating behaviours and their relationships with weight status have been reported in a number of populations globally. The Generation R Study found increased uncontrolled responses to food and food enjoyment to be associated with increased BMI in 4-year-old Dutch children [3]. The GUSTO study found a desire to drink and food enjoyment to be associated with increased weight status in 5 and 6 years old [4]. Finally the PEACHES and TEDS study were combined to show the impact of eating behaviours on child weight status at 7–9 years and 9–12 years old, respectively. Enjoyment of food, desire to drink and food responsiveness were associated with overweight in children [5]. Understanding the early life factors that affect these behaviours therefore may help us identify areas for intervention to curb the progression of overweight and obesity in children.

Breastfeeding is the optimum method of infant feeding as it has a plethora of short and long-term benefits for mothers and children. Therefore, the World Health Organisation (WHO) recommends exclusive breastfeeding for 6 months to all women globally, and to continue breastfeeding for minimum 2 years in conjunction with the introduction of safe, complimentary foods [6]. Despite this, in the WHO European region, from 2006 to 2012, only 25% infants were breastfed for 6 months, compared to a global rate of 40% [7]. Ireland has the lowest breastfeeding rates in this region in 2017 with 46.2% of infants not being breastfed, and similarly lowest rates of infants exclusively breastfed for 6 months at 10.5% [7].

A recent review by Ventura [8] discusses the importance of breastfeeding in establishing food preferences. Infants are born with an innate preference for sweet tastes, and are exposed to a variety of flavours while being breastfed, further influencing infant food preferences while weaning. Majority of previous studies have investigated food preferences, dietary intakes and their role in the aetiology of obesity. Child eating behaviours also hold importance, yet there is little research investigating breastfeeding and eating behaviours, such as food neophobia and picky eating [9–11]. Research regarding breastfeeding and picky eating remains equivocal. Studies such as the Generation R Study in the Netherlands and the Healthy Start Primary Intervention Study in Denmark have found a relationship in which prolonged breastfeeding is associated with decreased picky eating [10, 11]. However, the ALSPAC study found no evidence to suggest maintaining breastfeeding for a longer duration has any impact on fussy eating habits [12]. Similarly, McDermott et al. [13] found no association between breastfeeding practices and fussy eating habits in children aged 2–4 years. Research including behaviours other than food fussiness has suggested a protective effect of breastfeeding against obesogenic eating behaviours but calls for further analysis to confirm this association [14]. Clearly the aetiology of these behaviours is complex, requiring ongoing research for any consensus to be formed.

Parents have a major impact on a child’s eating behaviour through choosing the infant feeding practice, being the main providers of foods available and their interaction with their children during mealtimes [15]. A parent’s control over their child’s eating behaviour has previously been determined to occur via restriction and pressure feeding practices, which can have negative impacts on weight [16, 17]. Similarities between parent and adolescent eating behaviours have been observed in previous research [18, 19]. Whether parental eating behaviour is something a young child emulates when developing their own eating behaviour remains to be explored.

In order to address the existing gaps in the literature, the aim of this study is to investigate if a relationship exists between breastfeeding practices and maternal eating behaviours, with child eating behaviours in 5-year-old children. The author’s hypothesise that breastfeeding will encourage healthful eating behaviours, and that children will to some extent mirror their parent’s eating behaviours.

## Study design and methods

### Population

This is a secondary analysis of the ROLO RCT, a Randomised cOntrol trial of LOw glycaemic index (GI) diet in pregnancy to prevent recurrence of macrosomia (birthweight > 4 kg) in euglycemic women [20]. The ROLO RCT was carried out in the National Maternity Hospital (NMH), Dublin, Ireland. Ethical approval for this study was granted by the Ethics Committee of the NMH. The background, methods and results of this study have been published previously [21]. Participants consisted of secundigravida women, having previously given birth to a macrosomic baby, with no history of gestational diabetes or conditions requiring medication. Seven hundred and fifty-one participants were randomised to receive low GI dietary advice vs. usual care (no dietary advice) to reduce recurrence of macrosomia. Primary outcome was birthweight and secondary outcome was gestational weight gain and glucose intolerance. No difference was noted in birthweight, though significant maternal benefits were found in terms of less gestational weight gain and improved glucose tolerance [21]. These women were followed throughout pregnancy, birth and up to 5-year postnatally.

Mothers were contacted and invited to take part in the ROLO Kids follow-up study when their study child reached 5 years of age. Participants were eligible for the follow-up if their child had not yet reached 5 years and 6 months and gave fully informed written consent. Four hundred and three mother–child dyads returned for the 5-year follow-up. Of these, 230 pairs had data on mother and child eating behaviours and breastfeeding habits.

### Breastfeeding practices

Mothers reported breastfeeding duration retrospectively at 6 months, 2 years and 5 years postnatally. At each of these time points, mothers reported whether they had ever breastfed, and the duration in weeks that they breastfed for. A composite of answers given at each time point was used to dichotomise participants as breastfed vs. never breastfed as a measure of breastfeeding exposure, defined as ‘did you ever breastfeed your child’. A composite of breastfeeding duration was also determined for breastfeeding duration in weeks. Mothers that did not attend the 6-month follow-up but answered at 5 years could be included by using data reported at 5 years. If mothers had answered at an early time point, this was used instead of the duration reported at later time points to reduce memory bias.

### Assessment of eating behaviours

The Three Factor Eating Questionnaire (TFEQ) was used to determine maternal eating behaviours. This questionnaire was developed by Stunkard and Messick [22] and has been previously determined suitable in measuring eating behaviours [23, 24]. The TFEQ measures three different areas of behaviour: Uncontrolled Eating, Cognitive Restraint and Emotional Eating. Uncontrolled Eating represents the inability to resist foods when they are tempted, or if they are in the company of people enjoying a meal (Cronbach *α* = 0.854). Cognitive Restraint is indicative of individuals that make a conscious effort to avoid certain foods or not finish meals, with a goal of losing weight (Cronbach *α* = 0.708). Emotional Eating occurs when a mother eats more than usual due to generally negative emotions, e.g., anxiety (Cronbach *α* = 0.886). Mothers answered the TFEQ in relation to their own eating behaviour using a four-point Likert scale from definitely false to definitely true, almost never to almost always, unlikely to very likely, only at mealtimes to almost always and never to at least once a week, depending on the question. A higher score indicated the mother was more likely to experience this eating behaviour.

The Child Eating Behaviour Questionnaire (CEBQ) was used to describe child eating behaviours. The CEBQ was developed by Wardle et al. [25], ‘to provide a useful measure of eating style for research into the early precursors of obesity or eating disorders’. The CEBQ has been validated as an accurate measurement of child eating behaviours [26–28]. The CEBQ determines whether a child has a food approach behaviour or food avoidance behaviour. Both outcomes have four subsets. The food approach behaviour subset has been associated with increased weight status, and includes Food Responsiveness (inappropriate responses to the food environment around them, e.g., if allowed, my child would eat too much; Cronbach *α* = 0.811), Enjoyment of Food (is the child enjoying mealtimes and curious about food, e.g., my child is interested in food; Cronbach *α* = 0.888), Emotional Overeating (implies increased food intake during times of negative emotions, e.g., my child eats more when worried; Cronbach *α* = 0.691) and a Desire to Drink (implies the desire to have a drink frequently, sometimes sugar sweetened, e.g., my child is always asking for a drink; Cronbach *α* = 0.863) [25]. The food avoidance behaviour subsets include Slowness in Eating (taking a very long time to finish meals, e.g., my child takes more than 30 min to finish a meal; Cronbach *α* = 0.791), Satiety Responsiveness (ability to stop eating when the feeling of fullness occurs, e.g., my child gets full before they finish a meal; Cronbach *α* = 0.777), Food Fussiness (another term for picky eating, e.g., my child is difficult to please with meals; Cronbach *α* = 0.928) and Emotional Undereating (indicates a reduced intake of food during periods of emotional distress, e.g., my child eats less when angry; Cronbach *α* = 0.766). Each question contributes to one of the above behaviours. The mother completed the questionnaire using a five-point Likert scale from never to always; a higher score indicates the child is more likely to express this behaviour.

### Demographic information

Baseline characteristics were also recorded and adjusted for within the analysis. Maternal ethnicity and education level were recorded at recruitment into the ROLO study. Third-level education was dichotomised into those with or without third-level education (third level being universities, institutes of technology and colleges of education). Maternal and child BMI (kg/m^2^) was determined using height and weight measured by trained research nutritionists at the 5-year-old follow-up, rounded to the nearest 0.1 kg. The child’s exact age was calculated based on date of appointment. Birthweight was recorded during original ROLO pregnancy study. Type of childcare, such as family member at home, family member outside the home, creche, no childcare, etc., was recorded at the 5-year follow-up and noted as exposed or not exposed to childcare. Child birthweight centiles were calculated using Gestation Network’s Bulk Calculator 6.2.3 UK [29]. For the 5-year-old BMI centiles, they were calculated relevant to the 1990 UK reference data, using the Excel LMS Growth macro [30].

### Statistical analysis

Statistical Package for the Social Sciences (Version 24) was used for statistical analysis. Histograms were used for visual aid in determining normality in each of the variables—all eating behaviour variables were considered normally distributed and so parametric analysis measurements were used. The following descriptors were found to be skewed and so median and interquartile range were reported; maternal BMI, breastfeeding duration in weeks, child age, child birthweight centile, and child BMI centile at 5 years. Breastfeeding duration in weeks was log10 transformed prior to use in regression models.

The cohort was split into infants never exposed to breastfeeding and infants who had been exposed to any breastfeeding. Differences in child eating behaviours between these groups were measured using Student’s *t* tests. Multiple regression was carried out with breastfeeding exposure (ever breastfed vs. no breastfeeding) as a predictor of child eating behaviours, adjusted for RCT group, maternal education level, maternal BMI, childcare exposure (attended any form of childcare vs. no childcare) and child BMI centile at 5 years, which accounts for sex and age. Similar methods were used for determining associations with breastfeeding duration as the predictor of child eating behaviours, and maternal eating behaviours as the predictor of child eating behaviours. Spearman Rho was used to determine correlations between breastfeeding duration and child eating behaviours. Pearson correlations determined initial unadjusted associations between maternal and child eating behaviours. Those with a correlation *p* value of <0.05 were further analysed using multiple regression models. Models were adjusted for original RCT group, maternal education level, maternal BMI at 5 years, breastfeeding exposure, childcare exposure and child BMI centile at 5 years. Models with *p* < 0.05 were considered significant.

## Results

This analysis included 230 mother–child dyads of the ROLO 5-year follow-up; 105 were male (45.7%). There were 123 (53.5%) mothers in the intervention group of the RCT. Eighty-three per cent had achieved some third-level education and most children (91%) had been exposed to childcare.

Of those that returned at 5 years, 68.7% had breastfed their child. Of mothers that breastfed, median breastfeeding duration was 22 weeks (IQR: 33.00). Mean scores for childhood eating behaviours were as follows: 12.59 ± 4.05 for ‘food responsiveness’, 6.62 ± 1.99 for ‘emotional overeating’, 15.20 ± 2.91 for ‘enjoyment of food’, 8.05 ± 2.74 for ‘desire to drink’, 15.18 ± 3.24 for ‘satiety responsiveness’, 12.14 ± 3.18 for ‘slowness in eating’, 10.92 ± 3.36 for ‘emotional undereating’ and finally 18.41 ± 5.90 for ‘food fussiness’. For maternal eating behaviours, average scores were 18.17 ± 4.87 for ‘uncontrolled eating’, 6.67 ± 2.55 for ‘emotional eating’ and 13.93 ± 3.18 for ‘cognitive restraint’. Further details on demographics are reported in Table 1.

**Table 1:** ROLO^a^ participant characteristics, breastfeeding habits and mean eating behaviour scores at 5 years of age.

Maternal characteristics		
Age (years, mean, SD)	38.52	3.95
Ethnicity (White, Irish; *n*, %)	212	92.20
BMI^a^ at 5 years (kg/m^2^; median, IQR)	25.62	4.54
High education level^a^ (*n*, %)	178	83.20
ROLO RCT^a^ group—intervention (*n*, %)	123	53.50
Maternal eating behaviours—TFEQ		
Uncontrolled eating (mean, SD)	18.17	4.87
Emotional eating (mean, SD)	6.67	2.55
Cognitive restraint (mean, SD)	13.93	3.18
Breastfeeding habits		
Breastfeeding ever (yes, *n*, %)	158	68.70
Breastfeeding duration (weeks; median, IQR)	21.67	33.00
Child characteristics		
Sex (males; *n*, %)	105	45.70
Age (years; median, IQR)	5.12	0.15
Birthweight (kg; mean, SD)	4.00	0.44
Birthweight centile (median, IQR)	72.09	24.42
BMI at 5 years (kg/m^2;^ mean, SD)	16.18	1.33
BMI centile at 5 years (median, IQR)	61.87	25.34
Attended childcare (yes; *n*, %)	209	90.90
Child eating behaviours—CEBQ		
Food responsiveness (mean, SD)	12.59	4.05
Emotional overeating (mean, SD)	6.62	1.99
Enjoyment of food (mean, SD)	15.2	2.91
Desire to drink (mean, SD)	8.05	2.74
Satiety responsiveness (mean, SD)	15.18	3.24
Slowness in eating (mean, SD)	12.14	3.18
Emotional undereating (mean, SD)	10.92	3.36
Food fussiness (mean, SD)	18.41	5.90

*n* = 230.

^a^*ROLO* a randomised control trial of low glycaemic index diet to prevent macrosomia in euglycaemic women [22], *BMI* body mass index, *IQR* interquartile range, *High Education level* education from a higher education institute (universities, institutes of technology and colleges of education), *RCT* randomised control trial, *TFEQ* Three Factor Eating Questionnaire, *CEBQ* Child Eating Behaviour Questionnaire.

### Breastfeeding exposure and child eating behaviours

Children that had any exposure to breastfeeding were less likely to express a ‘desire to drink’ than those that had not been breastfed (7.77 vs. 8.65, *p* = 0.023) in unadjusted analysis. No other significant differences were identified. Further details can be seen in Table 2.

**Table 2:** Independent sample *t*-tests to determine differences in child eating behaviours at 5 years of age between breastfeeding exposure groups.

Child eating behaviour	Breastfed (*n* = 158)		Never Breastfed (*n* = 72)		*p*
	Mean	SD^a^	Mean	SD	
Food responsiveness	12.54	3.95	12.68	4.30	0.814
Emotional overeating	6.63	2.12	6.60	1.72	0.911
Enjoyment of food	15.26	2.67	15.06	3.39	0.653
Desire to drink	7.77	2.63	8.65	2.89	0.023
Satiety responsiveness	15.42	3.32	14.64	3.02	0.088
Slowness in eating	12.26	3.15	11.89	3.25	0.413
Emotional undereating	11.15	3.33	10.42	3.37	0.127
Food fussiness	18.27	5.74	18.71	6.28	0.604

Significant *p* value < 0.05.

^a^*SD* standard deviation.

Multiple regression analyses were carried out to adjust for confounding factors, including RCT group, maternal education level, maternal BMI, childcare exposure and child BMI centile at 5-year follow-up. The association between no breastfeeding exposure and increased ‘desire to drink’ remained in the adjusted analysis (*B* = −1.01, *p* = 0.022) (Table 3).

**Table 3:** Adjusted multiple regression models with exposure to breastfeeding as the predictor and child eating behaviours as the outcome variable.

Models^a^	B	*p*	Adjusted R^2^	95% confidence interval		*p*
				Lower	Upper	
Desire to drink						
Breastfeeding exposure	−1.008	0.022	0.025	−1.871	−0.144	0.088

Significant *p* < 0.05.

^a^Multiple regression analysis controlled for RCT group, maternal education level, maternal BMI, childcare exposure and infant BMI centile at 5 years. Child eating behaviours determined using CEBQ.

### Breastfeeding duration and child eating behaviours

Correlations between breastfeeding duration (weeks) and child eating behaviours were determined in unadjusted analysis. Breastfeeding duration was significantly associated with ‘food responsiveness’ (*B* = −0.21, *p* = 0.012), ‘desire to drink’ (*B* = −0.18, *p* = 0.030) and ‘emotional undereating’ (*B* = 0.20, *p* = 0.015). No other eating behaviours were significantly correlated with breastfeeding duration.

Significant correlations between breastfeeding duration (weeks) and child eating behaviours at 5 years of age were further investigated, adjusted for RCT group, maternal education level, maternal BMI, childcare exposure and child BMI centile at 5-year follow-up. ‘Food responsiveness’ was negatively, significantly associated with longer breastfeeding duration (*B* = −1.71, *p* = 0.003) (Table 4).

**Table 4:** Multiple regression analysis between breastfeeding duration (weeks) as predictor and child eating behaviours at 5 years of age as outcome variables.

	B	*p*	Adjusted R^2^	95% confidence interval		*p*
				Lower	Upper	
Child food responsiveness						
Breastfeeding duration^a^	−1.708	0.003	0.058	−2.817	−0.599	0.032
Child desire to drink						
Breastfeeding duration^a^	−0.744	0.057	0.012	−1.51	0.025	0.278
Child emotional undereating						
Breastfeeding duration^a^	0.950	0.046	0.058	−0.016	1.883	0.140

Multiple regression analyses controlled for RCT group, maternal education level, maternal BMI, childcare exposure and child BMI centile at 5 years. Significant *p* < 0.05, *n* = 150.

^a^Breastfeeding duration (predictor) measured in weeks. Child eating behaviours (outcome) determined using CEBQ.

### Maternal eating behaviours and child eating behaviours

In unadjusted analysis, maternal ‘uncontrolled eating’ was significantly associated with child ‘food responsiveness’ (*B* = 0.24, *p* < 0.001), ‘emotional overeating’ (*B* = 0.31, *p* < 0.001), ‘desire to drink’ (*B* = 0.14, *p* = 0.026) and ‘emotional undereating’ (*B* = 0.22, *p* = 0.001). Maternal ‘emotional eating’ was associated with ‘emotional overeating’ (*B* = 0.27, *p* < 0.001), ‘satiety responsiveness’ (*B* = 0.20, *p* = 0.002), ‘food fussiness’ (*B* = 0.18, *p* = 0.006) and ‘emotional undereating’ (*B* = 0.23, *p* = 0.001).

After adjusting models for confounders (RCT group, maternal education level, maternal BMI, childcare exposure and child BMI centile at 5 years), significant associations were found between maternal and child eating behaviours. Maternal ‘uncontrolled eating’ was positively associated with child ‘food responsiveness’ (*B* = 0.21, *p* < 0.001), ‘emotional overeating’ (*B* = 0.14, *p* = 0.005) and ‘emotional undereating’ (*B* = 0.14, *p* = 0.005). Maternal ‘emotional eating’ was positively associated with child ‘emotional overeating’ (*B* = 0.27, *p* < 0.001), ‘satiety responsiveness’ (*B* = 0.25, *p* = 0.009), ‘emotional undereating’ (*B* = 0.29, *p* = 0.004) and ‘food fussiness’ (*B* = 0.58, *p* = 0.001) (Table 5).

**Table 5:** Multiple regression analysis with maternal eating behaviours as predictor of child eating behaviours at 5 years of age.

Models	B	*p*	Adjusted R^2^	95% confidence interval		*p*
				Lower	Upper	
Child food responsiveness						
1. Uncontrolled eating	0.213	<0.001	0.067	0.096	0.330	0.003
Child emotional overeating						
1. Uncontrolled eating	0.141	0.005	0.052	0.044	0.239	0.011
2. Emotional eating	0.269	<0.001	0.081	0.155	0.383	0.001
Child desire to drink						
1. Uncontrolled eating	0.076	0.064	0.016	−0.005	0.157	0.162
Child satiety responsiveness						
1. Emotional eating	0.252	0.009	0.052	0.064	0.440	0.011
Child slowness in eating						
1. Emotional eating	0.155	0.102	0.037	-0.031	0.341	0.036
Child emotional undereating						
1. Uncontrolled eating	0.141	0.005	0.052	0.044	0.239	0.011
2. Emotional eating	0.289	0.004	0.054	0.094	0.483	0.010
Child food fussiness						
1. Emotional eating	0.577	0.001	0.049	0.235	0.920	0.014

Significance determined using individual multiple linear regression models adjusted for RCT group, maternal education level, maternal BMI at 5 years, breastfeeding exposure, childcare exposure and child BMI centile at 5 years. Significant at *p* < 0.05, *n* = 230. Maternal eating behaviours (predictor) determined using TFEQ. Child eating behaviours (outcome) determined using CEBQ.

## Discussion

This analysis found breastfeeding practices and maternal eating behaviours to be significantly associated with child eating behaviours at 5 years of age. Children that were breastfed at all were less likely to express a desire to drink sugary drinks. Children that were breastfed for longer were less likely to demonstrate food responsiveness and a desire to drink. We found that an increase in uncontrolled eating in mothers was associated with an increase in child food responsiveness and emotional overeating and undereating. Similarly, an increase in maternal emotional eating scores was associated with an increase in child emotional overeating and undereating, picky eating and responses to internal satiety cues.

### Breastfeeding and child eating behaviours

Sixty-eight per cent of women in this analysis had initiated breastfeeding with 37% of these women breastfeeding for the recommended 6 months. This is slightly higher than the WHO report, which reported an average of 23% of Irish mothers breastfed for 6 months [7]. The breastfeeding initiation rate is also slightly higher than that reported in 2017 which was 58% [31].

Previous studies including the Generation R Study and the Healthy Start Primary Intervention reported decreased fussy eating with longer breastfeeding duration [10, 11]. Our findings showed breastfeeding to have no association with fussy eating. Although in line with some previous studies, including the ALSPAC study [12], this was unexpected considering the importance of breastfeeding in developing the infants tastes and preferences for healthy foods [8]. Such contradictory evidence requires further studies to confirm the true association.

This is the first study to show that children that are not breastfed are more likely to have a constant want for drinks. Breastfeeding is thought to encourage infants to recognise internal satiety cues compared to bottle feeding, as breastfeeding is based on the infant’s needs rather than the timing considered appropriate by the caregiver [32]. Our findings that breastfeeding reduces the desire to drink may add to this discussion as to how infant feeding can impact food and drink choices throughout childhood.

Mother’s that breastfed for shorter durations perceived their children to inappropriately respond to the food around them and to have a greater desire for drinks. These behaviours have previously been associated with children having overweight and/or obesity [5, 11, 33, 34]. Other research has hypothesised that this relationship is due to a child’s ability to recognise a feeling of fullness, as breastfed infants learn to control their intakes better than formula fed infants [14, 35]. Furthermore, we found longer breastfeeding duration to be associated with children eating less in times of emotional distress. This may be a result of infants having improved control over their intakes and not considering food as a source of comfort.

The relationship between breastfeeding and behaviours of over consumption may also be attenuated by hormones present in breastmilk, including leptin, adiponectin and ghrelin [36–38]. These hormones are involved in appetite regulation and therefore are potentially linked to infant eating behaviours and infant weight status. The negative association between breastfeeding and inappropriate responses to food may be due to increased exposure to such hormones with longer breastfeeding, hence the infants learns appropriate response to hunger and satiation. The complexity in understanding the development of eating behaviours is clear as we discuss our own findings in comparison to existing literature.

These findings indicate that the relationship between breastfeeding and lower childhood obesity could be mediated by eating behaviours, as a desire to drink and food responsiveness have previously been linked to overweight and obesity during childhood [5, 33, 39, 40].

### Relationship between mother and child eating behaviours

It was found that maternal restrictive eating behaviours were not associated with her child’s perceived eating behaviour, however, uncontrolled eating and emotional eating both had significant associations with various child eating behaviours. Cognitive restraint is indicative of dieting, which may be a habit adopted at a later stage than 5 years, hence no association was observed.

Uncontrolled eating is the response to external food cues in mothers, showing a lack of self-control and high appetitive motivation [41]. We found maternal uncontrolled eating to be associated with offspring’s food responsiveness; mothers that find difficulty in resisting tempting foods had a child with similar obesogenic eating behaviours. Both uncontrolled eating and food responsiveness represent behaviours such as not being able to resist foods, excessive eating in times of distress and constant need to drink. These eating behaviours in children are indicative of an increased energy intake, particularly in our current obesogenic environment [42]. This indicates that children may emulate their mother’s impulsive reactions towards their food and drink environment. Our findings are supported by a previous study involving 8–12-year-old German children with a BMI centile ≥ 85%, which suggested a mother’s increased food intake behaviour may influence her child’s inability to self-regulate food impulses [43].

Such behaviours of increased desire to drink, inability to resist foods and excessive eating in emotional turmoil have been associated with increased child weight status in 7–12 years old across the UK, as shown by Webber et al. [5] using two cohorts: the PEACHES longitudinal and the TEDS cohort study. A mother’s eating behaviour could instil similar, unfavourable eating habits in her child, which result in increased energy intake, contributing to the development of overweight and obesity. It is clear that eating behaviours require further investigations to fully understand their development, considering parental impacts.

The complexity of eating behaviours is further shown in the contradictory relationships between maternal uncontrolled eating and child emotional undereating. Maternal uncontrolled eating was positively associated with a child eating less when sad/anxious. It is also associated with a child eating more in these times. In children, these behaviours are correlated with each other, which indicates children that have a tendency to overeat similarly undereat when distressed [25, 44]. The understanding that children often display both overeating and undereating in varying times of emotional distress may explain how the behaviour of emotional overeating in mothers is associated with the opposing behaviour of emotional undereating in their child. The emotional overeating subscale also includes eating in times of boredom, which also has some loading on the food responsiveness subscale. With this in mind, it may not be a true association between uncontrolled eating and emotional eating; it could potentially be mediated by eating due to boredom. There is possibility that this requires its own subscale to further elucidate childhood eating behaviours effectively.

Maternal emotional eating implies a greater energy intake than usual during times of great stress or negative emotion [23]. Our study found maternal emotional eating to be positively and significantly associated with increased eating in times of negative emotions. This may be simply explained by a mirror effect as infants observe mothers consuming excess, palatable foods when upset, causing the child to associate food with a comforting mechanism. However, mothers that tend to overeat in times of distress also perceived their children to eat less in times of stress, to be picky eaters and to have increased responses to a feeling of fullness. It is unclear how such contradictory results may occur when investigating perceived eating behaviours. This area warrants further detailed research.

In Ireland, strategies have been implemented to address the issue of childhood obesity, such as the *Safe*Food START Campaign [45]. Developing strategies that promote both breastfeeding and positive parental eating behaviours within the home could be a novel component of such public health campaigns with a goal of preventing childhood obesity. The START campaign does not currently address the topic of eating behaviours, nor do other similar public campaigns. Further research is necessary to determine if this is something that needs to be addressed.

### Strengths and limitations

This study is strengthened by the use of validated questionnaires to measure eating behaviours of mothers and children. To our knowledge, it is the first study to utilise results of the TFEQ and the CEBQ to analyse relationships between eating behaviours of mothers and children and overall eating behaviours and breastfeeding practices. The use of a composite value for breastfeeding duration also increases the validity of the recall and reduces memory bias, and allows inclusion of mothers that may not have reported breastfeeding at each follow-up. All children in this analysis were the second eldest sibling in the family, as per inclusion criteria of the original ROLO study, thus limiting bias of family dynamics which may affect child eating behaviours. This study is not without limitations. Including only second-born children may also be a limitation, as a parent’s perception of their first born child may be more concerned due to inexperience [46], hence giving different results in our parent-answered questionnaires. The CEBQ is a questionnaire regarding the child, intended to be reported by the primary caregiver. For this particular study, we only involved the mother. Therefore, if the mother is not the primary caregiver she is less likely to be fully aware of the child’s eating habits. To mitigate this, we adjusted analysis for childcare exposure. The mother may have perceived eating behaviours in a way that is similar to her own, creating a type of bias within the questionnaire scores. This cohort is well-educated which may not be fully representative of other populations. This is a common issue among interventional studies and is difficult to completely eliminate, which should be considered when interpreting the results. We made efforts to reduce such bias by adjusting for education level.

Our research furthers our understanding of the importance of both initiation and longevity of breastfeeding. This adds to the discussion surrounding the positives of breastfeeding. Women should be encouraged to breastfeed if at all possible, for as long as possible. It is vital that women are provided with adequately funded public services to avail of to encourage positive breastfeeding practices. Research investigating childhood eating behaviours is of clinical importance as we continue to describe the complexities involved, and how they can contribute to childhood health. Investigating these behaviours can lead to a better understanding of how they develop in children, and how they may contribute to the aetiology of childhood overweight and obesity, potentially leading to novel advances in tackling this current global epidemic.

## Conclusion

The findings of this study indicate that breastfeeding practices and positive maternal eating behaviours promote healthful child eating behaviours, known to influence child weight status. This further elucidates the benefits of breastfeeding, and how this extends right throughout childhood. It also adds to our understanding of parental involvement in the development of child eating behaviours.
